# Mapping Determinants of Hepatitis C Virus E1/E2 Transmembrane Interactions Using Intergenotypic Chimeras

**DOI:** 10.3390/v18060616

**Published:** 2026-05-28

**Authors:** Margherita Fanalista, Christina Holmboe Olesen, Rodrigo Velázquez-Moctezuma, Jens Bukh, Jannick Prentoe

**Affiliations:** 1 Copenhagen Hepatitis C Program (CO-HEP), Department of Infectious Diseases, Copenhagen University Hospital, 2650 Hvidovre, Denmark; margherita.fanalista@regionh.dk (M.F.); rodrigo.velazquez-moctezuma@sund.ku.dk (R.V.-M.); jbukh@sund.ku.dk (J.B.); 2 Copenhagen Hepatitis C Program (CO-HEP), Department of Immunology and Microbiology, Faculty of Health and Medical Sciences, University of Copenhagen, 2200 Copenhagen, Denmark; 3 Department of Biomedical Sciences (BMI), Division for Molecular and Translational Pharmacology, Faculty of Health and Medical Sciences, University of Copenhagen, 2200 Copenhagen, Denmark

**Keywords:** HCV, E1/E2 glycoproteins, transmembrane domains, viral entry, glycoprotein assembly, membrane interactions

## Abstract

Hepatitis C virus (HCV) infection remains a major global health burden, and no vaccine preventing chronic infection is available. The envelope glycoproteins, E1 and E2, form a complex essential for viral entry; however, the mechanisms governing E1/E2 assembly and stability remain incompletely defined. Here, we investigated the role of the E1/E2 transmembrane (TM) regions in HCV infectivity using chimeras of JFH1-based recombinants with isolate-specific Core-NS2 sequences in which the C-terminal TM domains of E1 (TME1), E2 (TME2), or both (TME1E2) from the H77 isolate (genotype 1a) replaced those of isolates representing genotypes 1–6. We further introduced the TM domains of S52 (genotype 3a) or J6 (genotype 2a) into H77 and included reciprocal swaps between J6 and S52. Most TM-swap chimeras displayed impaired infectivity; however, serial passaging led to partial recovery associated with adaptive mutations in E1/E2 mapping not only to the C-terminal TM regions but also to the E1 stem and the internal E1 TM region (iTME1). Extending the TME1 swap to include upstream α-helical segments improved infectivity in selected chimeras, whereas inclusion of iTME1 abolished infectivity. These findings support functional interactions between membrane-associated regions of E1/E2 and their ectodomains and highlight their relevance for E1/E2-based HCV vaccine design.

## 1. Introduction

About 50 million people are chronically infected with the hepatitis C virus (HCV) with increased risk of developing life-threatening cirrhosis and hepatocellular carcinoma [1,2,3]. Treatment of chronic infection with combinations of direct-acting antivirals (DAAs) targeting NS3, NS5A, and NS5B achieves cure rates exceeding 90% [4,5,6]. Nevertheless, global HCV elimination will require a vaccine that prevents the ~1 million new chronic infections annually [7,8]. However, this goal is hampered by numerous challenges, such as high viral genetic variability and limited understanding of how to recapitulate the native structure and assembly of HCV glycoproteins to induce neutralizing antibodies [9,10].

HCV is an enveloped positive-stranded RNA virus and the prototype member of the *hepaciviruses* group [11]. It is classified into 8 genotypes and more than 90 subtypes [12], with genotypes 1, 2, and 3 accounting for the majority of infections worldwide [4,13,14]. The HCV genome is about 9.6 kb in length and is translated into a single polyprotein, which is processed into ten viral proteins [15]. The viral envelope glycoproteins, E1 and E2, assemble as a non-covalent homodimer of E1/E2 heterodimers [16], anchored in the viral envelope lipid bilayer via transmembrane domains at the C-termini of E1 (TME1) and E2 (TME2) [16,17]. These TM domains are predicted to be around 30 amino acids long and start at positions 351 and 718, respectively (H77 reference sequence, GenBank AF009606; GenBank, Bethesda, MD, USA) [16,18]. Importantly, TME1 and TME2 likely function not only as membrane anchors, but also form a central interface for heterodimerization of E1 and E2, as truncation, replacement, or mutation, particularly of charged residues in the TME1 N-terminus, disrupts E1/E2 complex formation [19].

Besides TME1 and TME2, an additional hydrophobic domain has been described within the E1 ectodomain; however, soluble E1 protein can be generated by removal of the C-terminal domain alone [20]. Interestingly, during the execution of this study, we showed that in the full E1/E2 homodimeric complex, the C-terminal TMs combine with this internal E1 TM domain (iTME1; 260-290) to form a six-helix bundle, as also suggested in a recent prediction of the membrane-interacting parts of envelope proteins of HCV [21]. Furthermore, we found that upstream of TME1 sits an amphipathic region that ends at residue 329, followed by an α-helical segment extending to residue 308 [16]. However, the resolution in the TM domains was insufficient to address whether specific TM-packing interactions might occur within the membrane. Understanding the extent of these TM–TM interactions and their role in viral infectivity is essential for understanding the mechanisms underlying E1/E2 assembly. Moreover, clarifying how TME1 and TME2 contribute to heterodimerization is not only key for unraveling HCV entry but also highly relevant for vaccine design. Indeed, characterizing their role could inform the development of stabilized immunogens that preferentially present conserved neutralization sites, such as antigenic site 412 in E2 [22], and elicit broadly protective antibody responses [23,24,25].

In this study, we investigated the role of E1/E2 transmembrane domains in HCV infectivity by generating JFH1-based Core–NS2 chimeras carrying intergenotypic TM swaps across isolates from genotypes 1–6. We assessed how TME1, TME2, and combined swaps influence viral infectivity, examined the emergence of adaptive mutations in long-term cultures, and tested whether upstream stem regions or iTME1 contribute to TM compatibility. Through this approach, we aimed to define how transmembrane and adjacent segments regulate E1/E2 heterodimerization. Altogether, our results reveal genotype-specific constraints on TM compatibility and identify adaptive substitutions and stem helices that modulate E1/E2 function, indicating a mechanism of E1/E2 heterodimerization which relies on interactions of residues within TM helixes extending beyond the canonical C-terminal TM domains.

## 2. Materials and Methods

### 2.1. Cell Culture

Huh7.5 human hepatoma cells were cultured in Dulbecco’s Modified Eagle Medium (Invitrogen, Waltham, MA, USA) containing 10% heat-inactivated fetal bovine serum and 1% Penicillin-Streptomycin solution (Pen/Strep P4333, Sigma-Aldrich, Saint Louis, MO, USA). Cells were cultured in a dry incubator at 37 °C with 5% CO_2_ and split every 2–3 days.

### 2.2. Cloning

TM-swapped HCV-encoding plasmids were generated using the In-Fusion Kit (Takara Bio, Kusatsu, Shiga, Japan) from JFH1-based recombinants expressing Core–NS2 of isolates H77 [26], TN [27], J6 [28], S52 [29], ED43 [26], SA13 [30], or HK6a [31] (Sequences correspond to Genbank: EU363761, HQ852453, AF177036 (Core-NS2 ofJ6CF) with the rest from AB047639 (no consensus genbank entry exists for J6/JFH1), EU204645, EU363758, FJ393024, or FJ230883, respectively). To generate chimeras TME1 and TME2 regions were swapped at positions 351–383 and 718–746, respectively, based on the H77 reference sequence (GenBank: AF009606). For H77 TM swaps of JFH1-based J6 and S52 recombinants, additional swaps were introduced encompassing upstream extensions of TME1 (residues 342–350, 329–350, or 308–350) and/or the internal E1 transmembrane segment (iTME1; residues 260–290). Adaptive mutations were cloned using the QuikChange Lightning Multi-Site-Directed Mutagenesis kit (Agilent, Santa Clara, CA, USA). Plasmid DNA sequences were verified by Sanger sequencing (Macrogen Europe, Amsterdam, The Netherlands).

### 2.3. Transfection of Huh7.5 Cells

Huh7.5 cells were seeded at 4 × 10^5^ cells/well in 6-well plates 24 h prior to transfection. Plasmids encoding full-length JFH1-based HCV Core-NS2 recombinants and the corresponding TM swap chimeras were linearized with XbaI (Thermo Fisher Scientific, Waltham, MA, USA) and in vitro transcribed using a T7 RNA polymerase kit (Promega, Madison, WI, USA). RNA transcripts were transfected into Huh7.5 cells using lipofectamine 2000 (Invitrogen). After 6 h, cells from each transfection were trypsinized and redistributed into four wells of separate 48-well plates, corresponding to supernatant collection at 24, 48, 72, and 96 h post-transfection. Supernatants were then harvested at the indicated time points, sterile-filtered, and stored at −80 °C. Viral spread was monitored by immunostaining as described [29], using anti-NS5A 9E10 monoclonal antibody and secondary Alexa Flour 488 goat anti-mouse IgG (Invitrogen). Virus titers were determined as previously described [27,29]. Briefly, Huh7.5 cells were seeded at 7 × 10^3^ cells/well in 96-well plates. The day after, the collected supernatants were diluted in medium, added to the cells, and incubated for 48 h at 37 °C. Infected cells were fixed with methanol, then stained with anti-NS5A 9E10 monoclonal antibody [28] and secondary ECL anti-mouse IgG horseradish peroxidase (HRP)-linked whole antibody (NA931V, Amersham Biosciences, Little Chalfont, UK), followed by DAB staining. Focus-forming units (FFUs) were counted with the ImmunoSpot series 5 UV analyzer (CTL Europe GmbH, Bonn, Germany) [32]. Graphs displaying viral titers were generated using GraphPad Prism (v10.4.1).

### 2.4. Long-Term Culture Adaptations

Huh7.5 cells transfection with RNA transcripts from full-length JFH1-based HCV recombinants that exhibited viral attenuation was performed as above. Cells were passaged every 2–3 days, and viral spread was monitored by immunostaining as described [29], using the anti-NS5A 9E10 monoclonal antibody followed by Alexa Fluor 488-conjugated anti-mouse secondary antibody (Invitrogen, Waltham, MA, USA). Once viral spread reached approximately 80%, culture supernatants were collected, and viral RNA extracted. Mutations in the envelope glycoproteins were identified by nested RT-PCR of viral RNA, as described [29,30], followed by direct Sanger sequencing of the envelope proteins (Macrogen Europe, Amsterdam, The Netherlands).

## 3. Results

### 3.1. JFH1-Based Core-NS2 Strain-Specific HCV Recombinants Attenuated by H77 TM Domain Swaps (TM Chimeras)

**Figure 1:**
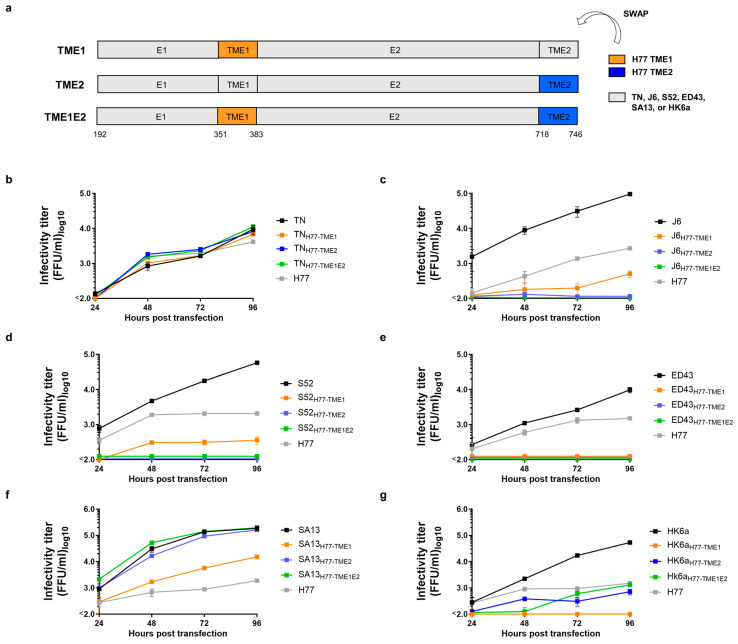
Swapping the TM domains of H77 E1 and/or E2 into different JFH1-based genotype 1-6 Core-NS2 recombinants has divergent attenuating effects. (**a**) We generated JFH1-based HCV recombinants with Core-NS2 from isolates TN(1a), J6(2a), S52(3a), ED43(4a), SA13(5a), or HK6a(6a), in which either TM domain of E1 (TME1), E2 (TME2) or both (TME1E2) were swapped with the corresponding H77 (genotype 1a) sequences. (**b**–**g**) In vitro transcribed RNA from H77 TM swap chimeras was transfected into Huh7.5 cells, and supernatants were collected at 24, 48, 72, and 96 h post-transfection. Viral infectivity at each time point was determined by infectivity titration in Huh7.5 cells. HCV-positive FFU were counted with ImmunoSpot series 5 UV analyzer (CTL Europe GmbH). Assay cut-off was 100 FFU/mL. Error bars represent the standard deviation of three technical replicates. Viral infectivity titration data summary and statistical testing can be found in Table 1.

We used JFH1-based HCV recombinants with Core-NS2 from isolates TN(1a), J6(2a), S52(3a), ED43(4a), SA13(5a), or HK6a(6a), to generate chimeric viruses in which either the C-terminal TME1, TME2 or both (TME1E2) were replaced with isolate H77(1a) sequences (Figure 1a). To test effects on viability for these H77 TM swap chimeras, we transfected Huh7.5 cells with in vitro transcribed RNA and collected cell supernatants at 24, 48, 72, and 96 h post-transfection. Viral titers of the supernatants were assessed by infection of naïve Huh7.5 cells. The attenuating effect varied across the tested genotype isolates. Swapping any combination of H77 TMs into TN, a closely related genotype 1a isolate which differs from H77 in the TM sequences at only two positions (I359M and A744V), did not cause attenuation (Figure 1b).

However, TME1, TME2, or TME1E2 H77 swaps into J6, S52, and ED43 caused a drastic reduction in viral infectivity, with only J6_H77-TME1_ and S52_H77-TME1_ showing detectable titers (Figure 1c–e). Swapping H77 TMs into the SA13 isolate had limited impact: SA13_H77-TME2_ and SA13_H77-TME1E2_ maintained titers comparable to the parental virus, whereas titers of SA13_H77-TME1_ showed a 12-fold reduction relative to SA13 but remained 8-fold higher than H77 (Figure 1f). In the HK6a isolate, the H77 TME1 swap completely abrogated infectivity, the TME2 swap reduced the titer ~75-fold, and replacement of both TME1 and TME2 yielded a titer comparable to H77 (Figure 1g). Overall, our data supports an interplay between the canonical C-terminal TMs of E1 and E2, but also point to the existence of additional interactions with regions outside of the TMs.

### 3.2. Viral Spread of H77 TM Chimeras Was Rescued in Long-Term Cultures

**Figure 2:**
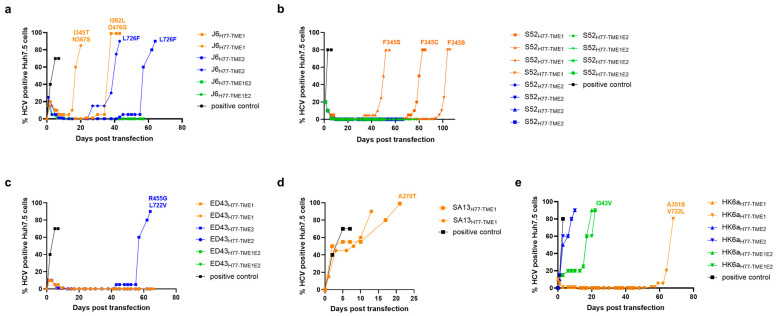
H77 TM swap chimeras acquire envelope proteins substitutions in long-term culture. (**a**–**e**) In vitro transcribed HCV RNA of H77 TM swap chimeras was transfected into Huh7.5 cells for long-term culture. Either H77 or S52 were used as positive control to assess viral spread. Viral spread at each cell passage was monitored by immunostaining with anti-HCV NS5A antibody, 9E10. Cultures were closed following negative HCV-specific staining for two consecutive weeks. When viral spread reached >80% HCV positive cells, cell supernatant was collected, and viral RNA was extracted. Putative adaptive mutations in envelope protein sequences were assessed by nested RT-PCR of viral RNA, followed by direct Sanger sequencing.

To study whether the attenuated H77 TM swap chimeras could adapt by passage in cell culture, we transfected in vitro transcribed RNA of these recombinants and monitored virus spread in long-term culture adaptations. Once the virus was able to spread to more than 80% of the culture, E1E2 from adapted viruses were sequenced for identification of adaptive mutations. J6_H77-TME1_ and J6_H77-TME2_ attenuation was rescued in long-term culture, whereas J6_H77-TME1E2_ attenuation persisted. Sequencing E1E2 from adapted J6_H77-TME1_ revealed the putative adaptive substitutions I262L in E1 and D476G in E2 in the first adaptation (day 43), and I345T in the stem of E1 together with N367S in TME1, in the second adaptation (day 20) (Figure 2a). Similar analysis of adapted J6_H77-TME2_ revealed the putative adaptive substitution L726F in TME2, in both independently adapted viruses (day 64 and day 43, respectively) (Figure 2a).

S52_H77-TME1_ acquired a single substitution at position F345 in the stem of E1 in three out of four independent long-term culture adaptations (day 55, day 85 and day 106). Conversely, we were unable to adapt S52_H77-TME2_ and S52_H77-TME1E2_ (Figure 2b). Of the six ED43 chimeras subjected to long-term adaptation, only one ED43_H77-TME2_ culture exhibited viral spread, and sequencing analysis revealed the substitution R455G in E2 together with L722V in TME2 (day 64) (Figure 2c). SA13_H77-TME1_ (the only attenuated TM-swap chimera from the SA13 isolate), spread in both long-term culture adaptations (day 13 and day 21). The substitution A270T in E1 emerged in one adaptation, while no changes were detected in the other (Figure 2d). Surprisingly, HK6a_H77-TME2_ spread after a week in culture without acquiring adaptive mutations in the envelope proteins. However, HK6a_H77-TME1_ attenuation was rescued in one long-term culture, with adaptive substitutions A351S and V722L occurring in TME1 and TME2 (day 68), respectively. Finally, analysis of HK6a_H77-TME1E2_ samples after viral spread revealed the emergence of the substitution I343V, in the E1 stem, in one of two adaptation cultures (day 22), while the other adaptation displayed no sequence variation (Figure 2e).

### 3.3. Chimeras with J6 and S52 TM Swaps

**Figure 3:**
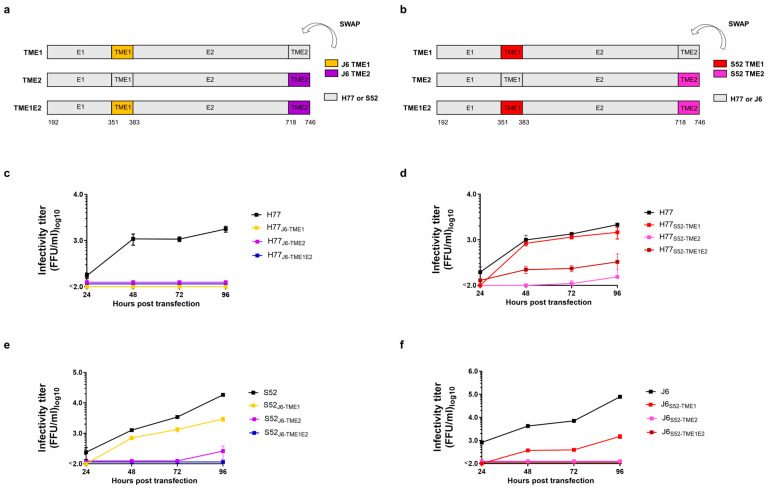
Inserting the TM domains of J6 and S52 into H77, J6, or S52 recombinants has isolate-dependent effects on viral infectivity. (**a**,**b**) We generated JFH1-based HCV recombinants with Core-NS2 from isolates H77(1a), J6(2a), or S52(3a), in which either TME1, TME2 or TME1E2 were swapped with the corresponding J6 or S52 sequence. (**c**–**f**) In vitro transcribed RNA from J6 and S52 TM swap chimeras was transfected into Huh7.5 cells and supernatants were collected at 24, 48, 72, and 96 h post-transfection. Viral infectivity at each time point was determined by infectivity titration in Huh7.5 cells. HCV-positive FFU were counted with ImmunoSpot series 5 UV analyzer (CTL Europe GmbH). Assay cut-off was 100 FFU/mL. Error bars represent the standard deviation of three technical replicates. Viral infectivity titration data summary and statistical testing can be found in Table 1.

To examine the effect of TM swaps in the context of the H77 JFH1-based Core-NS2 recombinant, we generated chimeras of H77 containing the C-terminal TMs domains of isolates J6 or S52. In addition, we generated recombinants for which the TMs had been swapped between the J6 and S52 isolates (Figure 3a,b). We tested viral titers from supernatants collected from Huh7.5 cells transfected with in vitro transcribed RNA and observed attenuation of H77 bearing TME1, TME2, or TME1E2 from isolate J6 (Figure 3c).

In contrast, H77 chimeras with S52 TMs retained infectivity: H77_S52-TME1_ yielded titers comparable to the parental virus, while H77_S52-TME2_ and H77_S52-TME1E2_ exhibited a 14- and 7-fold reduction (Figure 3d), respectively. Swapping J6 TMs into S52 caused complete attenuation of S52_J6-TME1E2_, a 77-fold reduction in the S52_J6-TME2_ chimera, but with a less pronounced effect in S52_J6-TME1_, with a 6-fold reduction in infectivity (Figure 3e). Swapping S52 TMs into the J6 isolate resulted in complete attenuation of J6_S52-TME2_ and J6_S52-TME1E2_. J6_S52-TME1_ showed a 50-fold reduction in infectivity compared to parental J6 (Figure 3f). Thus, our data further supported the existence of additional TM interactions with regions outside of the C-terminal TMs.

### 3.4. Viral Spread of J6 and S52 TM Chimeras Was Rescued in Long-Term Cultures

**Figure 4:**
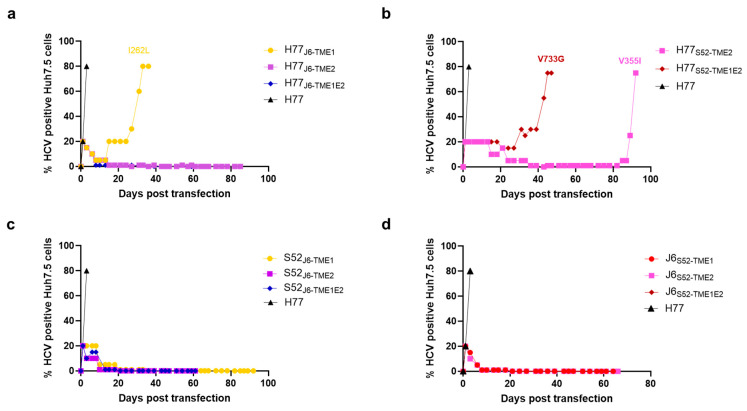
J6 and S52 TM-swapped H77 recombinants acquire envelope substitutions in long-term culture. (**a**–**d**) In vitro transcribed HCV RNA of J6 and S52 TM swap chimeras was transfected into Huh7.5 cells for long-term culture. The H77 Core-NS2 recombinant was used as a positive control to assess viral spread. Viral spread at each cell passage was monitored by immunostaining with anti-HCV NS5A antibody, 9E10. Cultures were closed following negative HCV-specific stainings for two consecutive weeks. When viral spread reached >80% HCV positive cells, the cell supernatant was collected, and viral RNA was extracted. Putative adaptive mutations in envelope protein sequences were assessed by nested RT-PCR of viral RNA, followed by direct Sanger sequencing.

The attenuated J6 and S52 TM swap chimeras were subjected to one long-term culture adaptation in Huh7.5 cells. Viral spread was monitored over time by immunostaining, and once infection reached 80% of the cell culture, E1E2 regions were sequenced to identify adaptive mutations. Among the attenuated H77 viruses carrying J6 TMs, only H77_J6-TME1_ spread, acquiring the single putative adaptive substitution I262L in E1 at day 36 post-transfection (Figure 4a). Since H77_S52-TME1_ produced titers comparable to parental H77 (Figure 3d), it was not subjected to adaptation. In contrast, both H77_S52-TME2_ and H77_S52-TME1E2_ spread in long-term culture, acquiring V355I in TME1 at day 92 and V733G in TME2 at day 45, respectively (Figure 4b). Surprisingly, all J6 chimeras carrying S52 TMs, as well as the reciprocal S52 chimeras carrying J6 TMs, had lost the ability to spread in long-term cultures (Figure 4c,d). This was despite the fact that J6_S52-TME1_ and S52_J6-TME1_ exhibited relatively high infectivity titers in the initial 4-day transfections (Figure 3e,f).

### 3.5. Observed E1 and E2 Substitutions Specifically Increase Viral Infectivity of TM-Swap Chimeras

**Figure 5:**
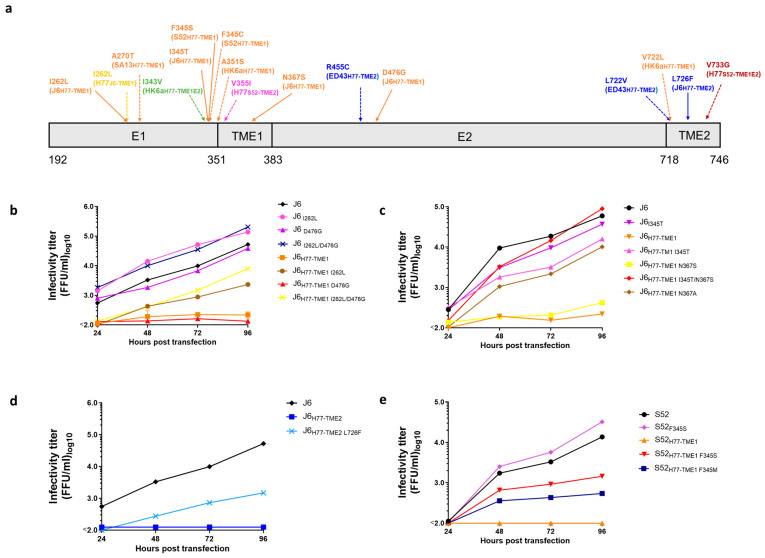
Envelope protein substitutions increase viral infectivity and compensate for H77 TM swap chimeras. (**a**) Envelope substitutions emerged during long-term culture of HCV TM swap chimeras of Core-NS2 recombinants bearing TM domains from isolates: H77 TME1 swap (orange); H77 TME2 swap (blue); H77 TME1E2 swap (green); J6 TME1 swap (yellow); S52 TME2 (pink); or S52 TME1E2 swap (brown). Continuous arrows indicate mutations confirmed to be TM-swap adaptive in reverse genetics infectivity assays, while dashed arrows indicate mutations not tested. The regions are numbered according to H77 abs. ref. (GenBank #AF009606). (**b**–**e**) Envelope protein substitutions found in long-term culture adaptations were cloned by site-directed mutagenesis into the corresponding H77 TM swap recombinant and the parental non-TM-swapped recombinant. In vitro–transcribed RNAs were transfected into Huh7.5 cells with lipofectamine, and supernatants were collected at 24, 48, 72, and 96 h post-transfection. These were used to infect naïve Huh7.5 cells for 48 h. HCV-positive foci were quantified using an ImmunoSpot Series 5 UV analyzer (CTL Europe GmbH). Assay cut-off was 100 FFU/mL. Error bars represent the standard deviation of three technical replicates. Viral infectivity titration data summary and statistical testing can be found in Table 2.

During long-term culture, several adaptive substitutions emerged in H77 TM chimeras of J6 and S52 recombinants, including I262L/D476G and I345T/N367S in J6_H77-TME1_, L726F in J6_H77-TME2_, and F345S in S52_H77-TME1_ (summary in Figure 5a; experimental data in Figure 2a,b). We tested these mutations to evaluate their potential adaptive and compensatory roles by introducing them into both the corresponding TM-swap chimeras and the parental non-TM-swapped recombinant. When more than one putative adaptive mutation was found in the same virus, mutations were cloned either alone or in combination to distinguish between their individual and combined effects. I262L/D476G and I345T/N367S changes increased J6_H77-TME1_ infectivity by 36-fold and 410-fold, respectively (Figure 5b,c). Notably, the I262L/D476G combination also caused a 4-fold increase in the infectivity of the parental J6 isolate (Figure 5b), though the effect was modest compared to the TM chimera. On the contrary, single mutations alone had a wide range of effects. While I262L and I345T alone increased J6_H77-TME1_ infectivity, D476G and N367S alone did not (Figure 5b,c). Because residue 367 is alanine in J6 but differs in the H77-derived TME1 of J6_H77-TME1_, we introduced alanine at this position (N367A) to test whether restoring the native residue would improve compatibility. Interestingly, the substitution N367A was enough to rescue infectivity of TM of J6_H77-TME1_, with only a 5-fold reduction in infectivity with respect to parental J6 (Figure 5c).

The substitution L726F showed a compensatory effect on J6_H77-TME2;_ however, it did not fully restore virus infectivity (Figure 5d). For S52_H77-TME1_, for which we observed multiple different changes at the same position, we chose to clone the most frequently observed substitution, F345S. When introduced in the TM chimera, the mutation partly compensated for the H77 TM swap, showing a ~14-fold increase, compared to a 2-fold increase in the parental S52 (Figure 5e). We also tested whether introducing a methionine at position 345, restoring the native H77 residue in the stem, would improve compatibility. However, the F345M substitution showed a smaller increase (Figure 5e), indicating that the mutation was not merely reestablishing an interaction that was affected by the chimeric nature of the protein.

### 3.6. Extending the Canonical H77 TME1 Swap Upstream Can Improve Infectivity of TM Swap Chimeras

**Figure 6:**
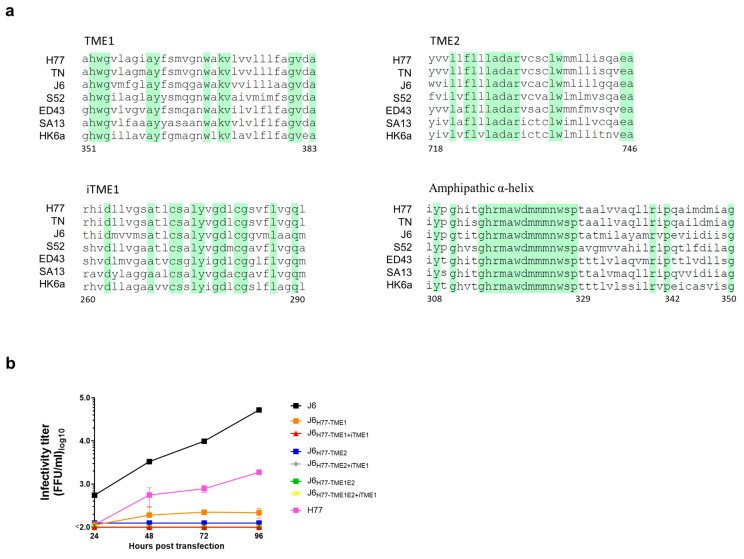
Sequence alignment of E1E2 membrane-associated regions and H77 iTME1 swap of JFH1-based J6 chimeras. (**a**) Sequence alignment of swap regions of isolates H77(1a), TN(1a), J6(2a), S52(3a), ED43(4a), SA13(5a) and HK6a(6a) (Clone Manager 9; Scoring matrix: BLOSUM 62): TME1 (381–383), TME2 (718–746), iTME1 (260–290) and membrane-associated amphipathic α-helices (308–350). Positions with 100% similarity are highlighted in green. (**b**) H77 TM-swap chimeras of J6 Core-NS2 recombinants were generated by replacing the internal E1 TM (iTME1) sequence with the corresponding H77 sequence. In vitro transcribed RNA was transfected into Huh7.5 cells using lipofectamine, and supernatants were collected at 24, 48, 72, and 96 h post-transfection. Harvested supernatants were used to infect naïve Huh7.5 cells for 48 h. HCV-positive foci were counted using an ImmunoSpot series 5 UV analyzer (CTL Europe GmbH). The assay cut-off was set at 100 FFU/mL. Error bars represent the standard deviation of three technical replicates. Viral infectivity titration data summary and statistical testing can be found in Table 1.

**Figure 7:**
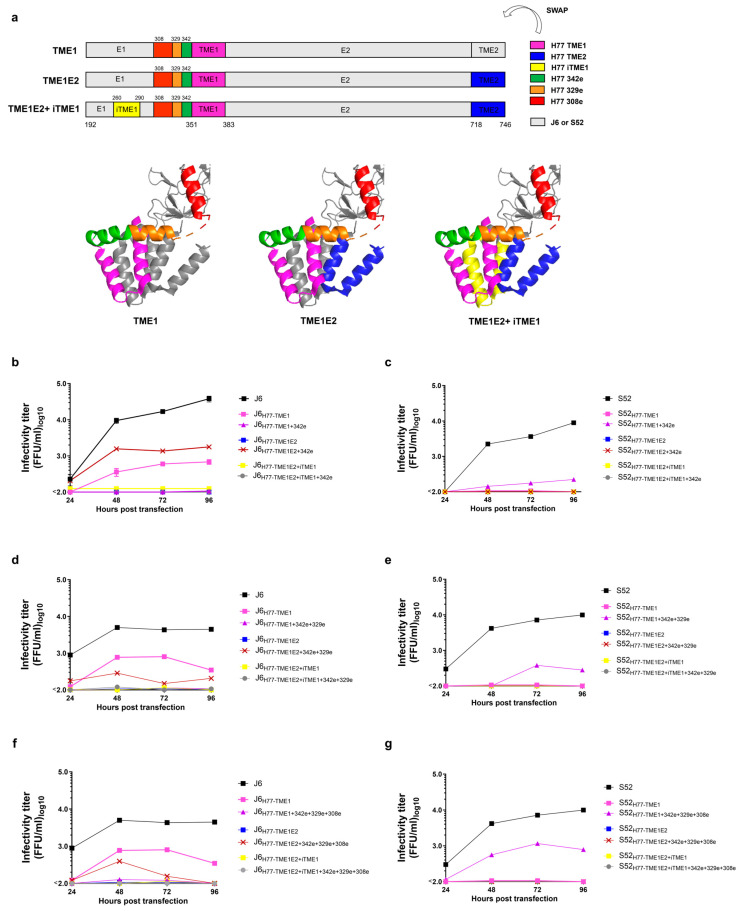
H77 TM-swap chimeras with H77 iTME1 and/or extended TME1 region swaps. (**a**) We generated H77 TM-swap chimeras for J6 or S52 Core-NS2 recombinants for H77 TME1, H77 TME1E2 and H77 TME1E2 + iTME1, in which the H77 TME1 swap regions were extended upstream till either position 342 (342e), 329 (329e) or 308 (308e). These domains are highlighted in the structural model of the six helix TM bundle of the E1/E2 homodimer complex structure (PDB 8rjj) as follows: TME1 (pink), TME2 (blue), iTME1 (yellow), extended upstream regions 342e (green), 329e (orange), and 308e (red). (**b**–**g**) In vitro transcribed RNA from H77 TM swap chimeras with additional iTME1 and/or extended TME1 regions were transfected into Huh7.5 cells and supernatants were collected at 24, 48, 72, and 96 h post-transfection. Viral infectivity at each time point was determined by infectivity titration in Huh7.5 cells. HCV-positive FFU were counted with ImmunoSpot series 5 UV analyzer (CTL Europe GmbH). Assay cut-off was 100 FFU/mL. Error bars represent the standard deviation of three technical replicates. Viral infectivity titration data summary and statistical testing can be found in Table 1.

Next, we examined the positions where adaptive mutations were acquired, comparing them to the TM bundle from our recently published full E1/E2 homodimer structure [16]. Here, we observed that, in TME1 swaps, these changes mapped to the internal E1 transmembrane segment (iTME1; residues 260–290) and to an amphipathic α-helix immediately upstream of TME1 (342e; residues 342–350), oriented perpendicular to TME1 along the membrane surface (Figure 5a and Figure 7a) [16]. However, this amphipathic helix continues after a kink at residue 329 (329e) and then continues into a second α-helical segment (308e) (Figure 6a and Figure 7a). 

Thus, to assess whether TME1, or TME1E2 swap attenuation was due to an incompatibility with these membrane-associated regions (i.e., iTME1 and amphipatic α-helix segments; Figure 6a), we did additional swaps. First, we swapped iTME1 alone, which did not improve viral titers in H77 TM chimeras of J6 recombinants (Figure 6b).We then tested elongated TME1 swaps in three H77 TM-swap chimeras of J6 or S52 recombinants: (i) TME1 alone, (ii) TME1E2, preserving the native TME1–TME2 pairing, and (iii) TME1E2 combined with iTME1, representing the complete swap of all TMs and membrane-associated α-helices. For each of these constructs, the H77 TME1 region was extended upstream to residues 342 (342e), 329 (329e), or 308 (308e) (Figure 7a). The TME1 upstream extension 342e caused a drop in infectivity of J6_H77-TME1_, but increased the infectivity of J6_H77-TME1E2_, which was highly attenuated and did not adapt in long-term culture (Figure 2a and Figure 7b). However, further extending the TME1 swap upstream to either 329e or 308e led to an infectivity loss (Figure 7d,f). In the S52 isolate, the 342e extension of TME1 slightly increased the infectivity of S52_H77-TME1_, but did not restore S52_H77-TME1E2_ infectivity (Figure 7c). When we extended the H77 swap region to encompass the 329e helix, this caused an increase in infectivity in S52_H77-TME1_ (Figure 7e), which further increased by extending the TME1 region with the 308e swap (Figure 7g). In contrast, adding iTME1 to the TME1E2 swap chimeras in combination with any upstream TME1 extension did not enhance titers (Figure 7b–g). Thus, extending the H77 TME1 swap to adjacent stem regions demonstrated that TME1 upstream amphipathic α-helices can modulate viral infectivity in an isolate-dependent manner, whereas iTME1 could not.

Together, these results support a model in which E1/E2 TM domains, in cooperation with neighboring stem elements, act as dynamic regulators of the E1/E2 complex, possibly with stem elements regulating TM-TM interactions, and iTME1 participating in distinct interactions critical for E1/E2 function. Surprisingly, swapping all membrane-associated regions did not result in viable viruses, perhaps indicating that some of these regions, at the interface between membrane-associated and ecto-domains, engage in multiple interactions. Overall, our study provides important insights for understanding HCV E1/E2 complex function, which could have important implications for future vaccine design.

## 4. Discussion

Despite advances in the structural characterization of the HCV E1/E2 glycoprotein complex, the molecular details of the TM interactions, particularly between the C-terminal regions of E1 (TME1) and E2 (TME2), remain to be fully elucidated [16,17,33]. Given that engineering of TM domains is a recognized strategy to stabilize viral glycoproteins for vaccine immunogen design, dissecting how HCV TMs regulate heterodimer stability provides a foundation for rational approaches to produce native-like E1/E2 immunogens [34]. Such stabilization could be critical to presenting conserved neutralization epitopes and thereby improve the ability of E1/E2-based vaccines to elicit broadly neutralizing antibodies [24,25,34,35,36].

In this study, we employed JFH1-based infectious HCV recombinants with genotype-specific Core-NS2 to generate chimeras carrying intergenotypic TM swaps between diverse HCV genotypes and further examined additional membrane-associated regions potentially involved in stabilizing the E1/E2 heterodimer. Based on the role of these regions as membrane anchors, we hypothesized that swapping either H77 (genotype 1a) TME1 or TME2 individually in different genotype isolates would reduce infectivity according to sequence compatibility between the TMs, while swapping both, thereby preserving the native H77 TME1 and TME2 pairing, might conserve infectivity. However, this pattern was observed only for the genotype 5a SA13 isolate (Figure 1f), indicating that TM–TM–mediated E1/E2 heterodimerization is influenced by additional determinants. To further assess genotype-specific effects on TM interactions, we performed reciprocal swaps between isolates J6 (2a) and S52 (3a), as well as swapping TMs from these isolates into H77 (the reverse swap from what we initially tested; Figure 3a,b). Reciprocal swaps between J6 and S52 confirmed strong isolate-specific constraints, with TME2 and TME1E2 replacements causing the most severe attenuation (Figure 3e–f). Notably, S52 TMs were better tolerated in H77 than J6 TMs (Figure 3c,d). Reverse swaps revealed asymmetric effects between isolates: H77 chimeras with S52 TMs maintained higher titers than reciprocal S52 chimeras with H77 TMs (Figure 1d and Figure 3d), and while J6 carrying H77 TME1 remained viable, the reciprocal H77 chimera with J6 TME1 was completely attenuated (Figure 1c and Figure 3c). A similar genotype-specific restriction was observed in a study assessing E1/E2 compatibility across genotypes 1a, 1b, and 2a using HCV pseudoparticles [37]. In that study, E2(1a) complemented E1(1b), whereas the reciprocal E1(1a)–E2(1b) pairing was non-infectious and could not be rescued by replacing E2(1b) TME2 with that of 1a to match genotype-specific TME1/TME2 pairs. Similarly, E2(1a) failed to complement E1(2a), and swapping E1(2a) TME1 with that of 1a did not restore infectivity [37]. These observations are consistent with our results showing that swapping both TME1 and TME2 from the same isolate does not necessarily lead to infectivity of HCV cell culture particles, pointing to additional determinants outside the C-terminal TM domains that govern E1/E2 heterodimerization and isolate-specific compatibility.

Long-term culture of attenuated TM swap chimeras revealed isolate- and genotype-specific patterns of adaptation, with putative compensatory mutations clustering in iTME1, the E1 stem, and the E1 and E2 C-terminal TM domains (Figure 5a). Functional testing of a subset of these mutations confirmed that they acted as compensatory changes for disrupted TM interactions. Specifically, I262L/D476G markedly enhanced J6_H77-TME1_ infectivity, whereas I345T/N367S restored infectivity to parental levels (Figure 5b,c), and L730F increased J6_H77-TME2_ infectivity (Figure 5d). Similarly, F345S substitution in S52_H77-TME1_ partially compensated for the H77 TM swap (Figure 5e). These findings in J6 and S52 chimeras demonstrate that adaptive mutations in E1 and E2 can compensate for altered TME1–TME2 interactions, potentially with residues I262 and I345 as critical determinants of E1/E2 heterodimerization. Interestingly, I262L has been reported to improve the infectivity of J6 lacking HVR1, implicating this residue in stabilizing E1/E2 structure [38]. We also previously observed the emergence of the I262L mutation in an HVR1-deleted J6 recombinant, associated with reduced sensitivity to antibody neutralization [39]. Additionally, I262A has been shown to alter HCV receptor usage by shifting HCV dependency for entry on claudin-1 to claudin-6 [40]. Together, these findings suggest that I262 might function as a key regulator of TM-dependent conformational changes that modulate receptor engagement and viral entry efficiency. While D476G alone did not enhance infectivity, its synergistic effect in combination with I262L may be linked to its location within HVR2 (residues ~460–485), a region frequently subject to sequence variation and implicated in modulating E1/E2 conformational dynamics [41,42]. Interestingly, D476G has previously been observed in human liver chimeric mouse infections and increased broad antibody resistance [43]. This points to a potential link between the HCV TMs and the recently described global E1/E2 conformational dynamics that govern antibody sensitivity and receptor dependency [44,45]. Additionally, mutation at position 367 has been reported to abolish infectivity while it does appear to preserve E1/E2 heterodimerization, whereas residue L726 within TME2 has been identified as critical for both E1/E2 heterodimerization and viral entry [46]. While we previously showed that mutating position I345 can enhance HCV viral fitness [39], the fact that two independent adaptations of distinct H77 TME1 chimeras (S52_H77-TME1_ and J6_H77-TME1_) acquired substitutions at this site suggests a key role for I345 in mediating H77 TME1–TME2 pairing. This is also supported by the observation that the effect on infectivity is modest on J6 and S52 parental viruses compared to the corresponding TM swap chimeras (Figure 5c,e).

Interestingly, the observed pattern of TM swap-related adaptation sites in E1 and E2 largely occurred at positions shown to interact with membranes in our recently reported cryo-EM structure of the full E1/E2 homodimeric complex [16]. Indeed, adaptive mutations clustered in iTME1 and in the E1 stem segment 342e, which we had found to form a membrane-associated amphipathic α-helix. Therefore, we tested whether these regions, as well as more upstream segments 329e and 308e (See Figure 7a for a visual representation demonstrating the rationale for why these junction sites were chosen), could modulate TM–TM interactions. In the J6 isolate, adding the H77 342e swap extension reduced titers of J6_H77-TME1_ but, importantly, increased the infectivity of J6_H77-TME1E2_, which was otherwise highly attenuated (Figure 7b). However, extending the swap further to include 329e or 308e caused a drop in infectivity (Figure 7d, f). In the S52 isolate, extending the H77 swap to 342e slightly increased infectivity of S52_H77-TME1_, with further improvements at 329e and the greatest effect at 308e (Figure 7c,e,g). However, none of these extensions enhanced the titers of the attenuated S52_H77-TME2_ or S52_H77-TME1E2_ chimeras. Notably, for H77 TM-swapped J6- and S52-based recombinants, incorporation of iTME1 into TME1E2, either alone or in combination with upstream extensions, did not enhance infectivity (Figure 7b–g), and in J6 recombinants, it reduced the infectivity of J6_H77-TME1_ (Figure 6b). Together, these findings support that membrane-associated α-helices immediately upstream of TME1 can modulate TM interactions and potentially contribute to E1/E2 heterodimerization, although their effects remain highly context dependent, indicating either that these helices at the membrane-surface interface are interacting both with the TMs and the ectodomains or are suggestive of the existence of additional TM interaction partners.

In summary, our study identifies TME1 and TME2 as key determinants of HCV infectivity, with domain swaps revealing asymmetric, isolate- or genotype-specific effects on TM-TM interactions. Adaptive changes clustered in iTME1 and TME1 adjacent segments, suggesting that stem–TM interactions regulate glycoprotein conformational dynamics. However, swapping the iTME1 always had a negative effect on infectivity, indicating perhaps that this segment is also involved in critical non-TM interactions. Our work has important implications for understanding E1/E2 function and dynamics and consequently also for rational vaccine design against this pervasive human pathogen.

## Figures and Tables

**Table 1:** Schematic overview of the tested intergenotypic TM-swap chimeras. Mean viral titers of each TM-swap chimera at 24, 48, 72, and 96 h post-transfection are presented as log_10_ (FFU/mL). Statistical significance was assessed by comparing titers to those of the corresponding parental viruses using Dunnett’s multiple comparisons test. *p* values are indicated as follows: *p* < 0.05 (*), *p* < 0.01 (**), *p* < 0.001 (***), *p* < 0.0001 (****), and not significant (ns). Data represent the mean of three technical replicates. Statistical analyses were performed using GraphPad Prism (v10.4.1).

	24 h		48 h		72 h		96 h	
TN_H77-TME1_	<2.0	ns	3.0	ns	3.2	ns	3.8	**
TN_H77-TME2_	<2.0	ns	3.2	ns	3.4	ns	3.9	ns
TN_H77-TME1E2_	2.0	ns	3.1	ns	3.4	ns	4.0	ns
J6_H77-TME1_	<2.0	ns	2.1	**	2.2	****	2.7	****
J6_H77-TME2_	<2.0	ns	2.0	**	<2.0	****	<2.0	****
J6_H77-TME1E2_	2.0	ns	<2.0	**	<2.0	****	<2.0	****
S52_H77-TME1_	<2.0	ns	2.5	****	2.5	****	2.6	****
S52_H77-TME2_	<2.0	ns	<2.0	****	<2.0	****	<2.0	****
S52_H77-TME1E2_	<2.0	ns	<2.0	****	<2.0	****	<2.0	****
ED43_H77-TME1_	<2.0	ns	<2.0	**	<2.0	****	<2.0	****
ED43_H77-TME2_	<2.0	ns	<2.0	**	<2.0	****	<2.0	****
ED43_H77-TME1E2_	<2.0	ns	<2.0	**	<2.0	****	<2.0	****
SA13_H77-TME1_	2.4	ns	3.2	*	3.8	****	4.2	****
SA13_H77-TME2_	2.9	ns	4.2	ns	5.0	***	5.2	ns
SA13_H77-TME1E2_	3.3	ns	4.7	ns	5.1	ns	5.3	ns
HK6a_H77-TME1_	<2.0	ns	<2.0	**	<2.0	****	<2.0	****
HK6a_H77-TME2_	2.0	ns	2.6	*	2.5	****	2.8	****
HK6a_H77-TME1E2_	<2.0	ns	2.0	**	2.8	****	3.1	****
H77_J6-TME1_	<2.0	ns	<2.0	****	<2.0	****	<2.0	****
H77_J6-TME2_	<2.0	ns	<2.0	****	<2.0	****	<2.0	****
H77_J6-TME1E2_	<2.0	ns	<2.0	****	<2.0	****	<2.0	****
H77_S52-TME1_	<2.0	ns	2.9	ns	3.1	ns	3.2	****
H77_S52-TME2_	<2.0	ns	<2.0	****	2.0	****	2.2	****
H77_S52-TME1E2_	<2.0	ns	2.3	****	2.3	****	2.5	****
S52_J6-TME1_	<2.0	ns	2.9	ns	3.1	****	3.5	****
S52_J6-TME2_	<2.0	ns	<2.0	*	<2.0	****	2.4	****
S52_J6-TME1E2_	<2.0	ns	<2.0	*	<2.0	****	<2.0	****
J6_S52-TME1_	<2.0	ns	2.6	****	2.6	****	3.2	****
J6_S52-TME2_	<2.0	ns	<2.0	****	<2.0	****	<2.0	****
J6_S52-TME1E2_	<2.0	ns	<2.0	****	<2.0	****	<2.0	****
J6_H77-TME1 + iTME1_	<2.0	ns	<2.0	****	<2.0	****	<2.0	****
J6_H77-TME2 + iTME1_	<2.0	ns	<2.0	****	<2.0	****	<2.0	****
J6_H77-TME1E2 + iTME1_	<2.0	ns	<2.0	****	<2.0	****	<2.0	****
J6_H77-TME1 + 342e_	<2.0	ns	<2.0	****	<2.0	****	2.0	****
J6_H77-TME1E2 + 342e_	2.3	ns	3.2	****	3.1	****	3.3	****
J6_H77-TME1E2 + iTME1 + 342e_	<2.0	ns	<2.0	****	<2.0	****	<2.0	****
S52_H77-TME1E2 + iTME1_	<2.0	ns	<2.0	****	<2.0	****	<2.0	****
S52_H77-TME1 + 342e_	<2.0	ns	2.2	****	2.2	****	2.3	****
S52_H77-TME1E2 + 342e_	<2.0	ns	<2.0	****	<2.0	****	<2.0	****
S52_H77-TME1E2 + iTME1 + 342e_	<2.0	ns	<2.0	****	<2.0	****	<2.0	****
J6_H77-TME1 + 342e + 329e_	<2.0	****	<2.0	****	2.1	****	2.0	****
J6_H77-TME1E2 + 342e + 329e_	2.2	****	2.5	****	2.2	****	2.3	****
J6_H77-TME1E2 + iTME1 + 342e + 329e_	<2.0	****	2.1	****	<2.0	****	2.0	****
S52_H77-TME1 + 342e + 329e_	<2.0	ns	<2.0	****	2.6	****	2.4	****
S52_H77-TME1E2 + 342e + 329e_	<2.0	ns	<2.0	****	<2.0	****	<2.0	****
S52_H77-TME1E2 + iTME1 + 342e + 329e_	<2.0	ns	<2.0	****	<2.0	****	<2.0	****
J6_H77-TME1 + 342e + 329e + 308e_	<2.0	****	2.1	****	2.1	****	<2.0	****
J6_H77-TME1E2 + 342e + 329e + 308e_	2.0	****	2.6	****	2.2	****	<2.0	****
J6_H77-TME1E2 + iTME1 + 342e + 329e + 308e_	<2.0	****	<2.0	****	<2.0	****	<2.0	****
S52_H77-TME1 + 342e + 329e + 308e_	2.0	ns	2.7	****	3.1	****	2.9	****
S52_H77-TME1E2 + 342e + 329e + 308e_	<2.0	ns	<2.0	****	<2.0	****	<2.0	****
S52_H77-TME1E2 + iTME1 + 342e + 329e + 308e_	<2.0	ns	<2.0	****	<2.0	****	<2.0	****

**Table 2:** Schematic overview of the tested intergenotypic TM-swap chimeras carrying adaptive mutations. Mean viral titers of each TM-swap chimera carrying adaptive mutations at 24, 48, 72, and 96 h post-transfection are presented as log_10_ (FFU/mL). Statistical significance was assessed by comparing titers of chimeras containing adaptive mutations to those of the corresponding TM-swap viruses (either J6_H77-TME1_, J6_H77-TME2_, or S52_H77-TME1_) using Dunnett’s multiple comparisons test. *p* values are indicated as follows: *p* < 0.01 (**), *p* < 0.001 (***), *p* < 0.0001 (****), and not significant (ns). Data represent the mean of three technical replicates. Statistical analyses were performed using GraphPad Prism (v10.4.1).

	24 h		48 h		72 h		96 h	
J6_H77-TME1 I262L_	<2.0	ns	2.6	ns	2.9	**	3.4	****
J6_H77-TME1 D476G_	2.1	ns	2.1	ns	2.2	ns	2.1	ns
J6_H77-TME1 I262L/D476G_	2.1	ns	2.6	ns	3.2	****	3.9	****
J6_H77-TME1 I345T_	2.5	ns	3.3	ns	3.5	ns	4.2	****
J6_H77-TME1 N367S_	2.1	ns	2.3	ns	2.3	ns	2.6	ns
J6_H77-TME1 I345T/N367S_	2.2	ns	3.5	ns	4.2	****	5.0	****
J6_H77-TME1 N367A_	<2.0	ns	3.0	ns	3.3	ns	4.0	****
J6_H77-TME2 L730F_	<2.0	ns	2.4	ns	2.9	****	3.2	****
S52_H77-TME1 F345S_	<2.0	ns	2.8	****	3.0	****	3.2	****
S52_H77-TME1 F345M_	<2.0	ns	2.6	**	2.6	***	2.7	****

## Data Availability

The original contributions presented in this study are included in the article. Further inquiries can be directed to the corresponding author.

## Abbreviations

The following abbreviations are used in this manuscript:

DAA	Direct-acting antivirals
FFU	Focus-forming units
HCV	Hepatitis C virus
HRP	Horseradish peroxidase
iTME1	Internal transmembrane region of E1
TM	Transmembrane region
TME1	C-terminal transmembrane region of E1
TME2	C-terminal transmembrane region of E2
TME1E2	C-terminal transmembrane regions of E1 and E2
